# Performance of Genotype Imputation for Rare Variants Identified in Exons and Flanking Regions of Genes

**DOI:** 10.1371/journal.pone.0024945

**Published:** 2011-09-19

**Authors:** Li Li, Yun Li, Sharon R. Browning, Brian L. Browning, Andrew J. Slater, Xiangyang Kong, Jennifer L. Aponte, Vincent E. Mooser, Stephanie L. Chissoe, John C. Whittaker, Matthew R. Nelson, Margaret Gelder Ehm

**Affiliations:** 1 Genetics, GlaxoSmithKline, Research Triangle Park, North Carolina, United States of America, King of Prussia, Pennsylvania, United States of America, and Stevenage, United Kingdom; 2 Department of Genetics, Department of Biostatistics, University of North Carolina, Chapel Hill, North Carolina, United States of America; 3 Department of Biostatistics, University of Washington, Seattle, Washington, United States of America; 4 Division of Medical Genetics, Department of Medicine, University of Washington, Seattle, Washington, United States of America; VU University Medical Center and Center for Neurogenomics and Cognitive Research, Netherlands

## Abstract

Genotype imputation has the potential to assess human genetic variation at a lower cost than assaying the variants using laboratory techniques. The performance of imputation for rare variants has not been comprehensively studied. We utilized 8865 human samples with high depth resequencing data for the exons and flanking regions of 202 genes and Genome-Wide Association Study (GWAS) data to characterize the performance of genotype imputation for rare variants. We evaluated reference sets ranging from 100 to 3713 subjects for imputing into samples typed for the Affymetrix (500K and 6.0) and Illumina 550K GWAS panels. The proportion of variants that could be well imputed (true r^2^>0.7) with a reference panel of 3713 individuals was: 31% (Illumina 550K) or 25% (Affymetrix 500K) with MAF (Minor Allele Frequency) less than or equal 0.001, 48% or 35% with 0.001<MAF< = 0.005, 54% or 38% with 0.005<MAF< = 0.01, 78% or 57% with 0.01<MAF< = 0.05, and 97% or 86% with MAF>0.05. The performance for common SNPs (MAF>0.05) within exons and flanking regions is comparable to imputation of more uniformly distributed SNPs. The performance for rare SNPs (0.01<MAF< = 0.05) was much more dependent on the GWAS panel and the number of reference samples. These results suggest routine use of genotype imputation for extending the assessment of common variants identified in humans via targeted exon resequencing into additional samples with GWAS data, but imputation of very rare variants (MAF< = 0.005) will require reference panels with thousands of subjects.

## Introduction

Imputation and analysis of untyped genetic variants provides a more comprehensive picture of genetic variation within a genomic region than analysis of only typed variants [1]. It has been a key tool contributing to the recent success of Genome-Wide Association Studies (GWAS). Genotype imputation methods combine haplotypes found in a study sample with the full haplotypes available in a more densely genotyped reference set to fill in missing genotypes. Imputation methods have been extensively evaluated for imputing the genotypes of HapMap SNPs into subjects with GWAS data [2]. The establishment of a resource for imputation is one of the key aims of the 1000 Genomes Project [3]. In addition, studies sequencing specific genes or the exome in thousands of subjects are available now [4], at higher depth which enables the calling of individual genotypes with error rates comparable with other genotyping methods. The variants identified from high depth sequencing of the exons and flanking regions of genes have a SNP density distribution quite different than that available for the HapMap and 1000 Genomes data where there are relatively few large gaps between variants, and variants have an average inter-SNP distance of 875 bp [5] and 200 bp [3], respectively. The variants identified in the coding regions of genes are concentrated in short regions within a gene interspersed with longer regions with no variants. Variants genotyped by next generation sequencing methods yield very rare heterozygous calls with higher confidence enabling novel variant identification. High depth sequence data for thousands of samples has resulted in high quality rare variant calls in minor allele frequency (MAF) ranges not seen with prior HapMap or 1000 Genomes efforts. These efforts not only focused on sequencing a smaller number of individuals but also used technologies with lower confidence in very rare heterozygous calls. As sequencing studies focusing on sequencing the exomes of genes are in progress, characterizing the performance of imputation methods for variants in the exons and flanking regions, especially for variants with MAF less than or equal to 0.05, will provide a comprehensive picture of the use of imputation to extend these association studies into additional samples with GWAS data. Until now, no other study has provided a summary of the performance of genotype imputation for variants with minor allele frequencies less than 0.01.

There are a number of methods providing genotype imputation including IMPUTE [6], MaCH [7], and BEAGLE [8]. All three methods have been extended to accommodate multiple GWAS platforms and reference panels with more than 1000 samples. We selected BEAGLE for our analyses. A comparison of imputation performance for minimac, http://genome.sph.umich.edu/wiki/minimac, (an extention of MaCH software which takes haplotypes as input) and BEAGLE, using data from chromosome 1, showed them to be similar. For this chromosome, there were 111 and 152 SNPs with estimated r^2^ greater than 0.7 using minimac and BEAGLE, respectively. The median true r^2^ for these sets of variants was 0.977 and 0.982 for minimac and BEAGLE, respectively (Figure S1). The corresponding mean genotype error rates were 0.011 and 0.0124 and the mean allelic error rates were 0.0055 and 0.0062, for minimac and BEAGLE, respectively.

We recently completed high depth sequencing (median depth of 27×) of the exons and flanking regions of 202 genes that are current or prospective drug targets in over 14000 samples, 12514 of European ancestry (confirmed by principal component analysis ), including population-based and case collections (described in the Information S1) for 12 diseases. Analyses of experimental duplicates and capillary sequencing for a subset of these samples yielded an overall heterozygote genotype error rate of 0.50%. Of these sequenced samples, 8865 have GWAS data, including the Affymetrix 500K (n = 3983), Affymetrix 6.0 (n = 573) and Illumina 550K (n = 4309) GWAS platforms. We used subsets of these samples in an evaluation where we partitioned sequenced samples into reference and “to-be-imputed” study sets and compared the imputed genotypes with genotypes derived from high depth sequence data from the DeepSeq Variant Set.

Our primary goal was to develop a strategy for genotype-phenotype analysis utilizing genotype imputation for this dataset. We characterized the performance of genotype imputation with reference panels ranging from 100 to 3713 subjects for variants distributed within the exons and flanking regions of genes. Our results show that genotype imputation into additional samples with GWAS data will increase the sample size available for genotype-phenotype analysis for common and moderately rare variants with performance depending on the reference panel size for very rare variants (MAF<0.005).

## Results

This evaluation focused on characterizing the performance of genotype imputation for reference panels of 100 to 3713 subjects and variants present in the exons and flanking regions of genes. As the DeepSeq Variant Set had high quality genotype calls derived from high depth sequence data for 8865 subjects, we were able to characterize genotype imputation performance for variants with minor allele frequencies less than 0.01. We first summarize how well the estimated r^2^, the ratio of the variance of the imputed allelic dosage and the variance of the true allelic dosage assuming Hardy- Weinberg equilibrium [7], correlates with true r^2^, squared correlation of the true allelic dosage and the imputed allelic dosage [7] based on non-integer dosages which incorporate uncertainty in the imputed genotypes, for all reference and study samples listed in Table 1.

**Table 1:** Data sets used for characterizing genotype imputation.

Platform	Total number of samples with sequence data	Reference set sizes evaluated	Study set size used
Affymetrix 500K	3983	100, 300, 600, 1200, 3713	270
Affymetrix 6.0	573	100, 300	270
Affymetrix 6.0	573	562	11
Illumina 550K	4309	100, 300, 600, 1200, 3713	270

We partitioned the total number of samples with sequence data into reference samples and “to-be-imputed” study samples. We used the reference panel samples to predict unobserved genotypes in the study sample.

**Figure 1:**
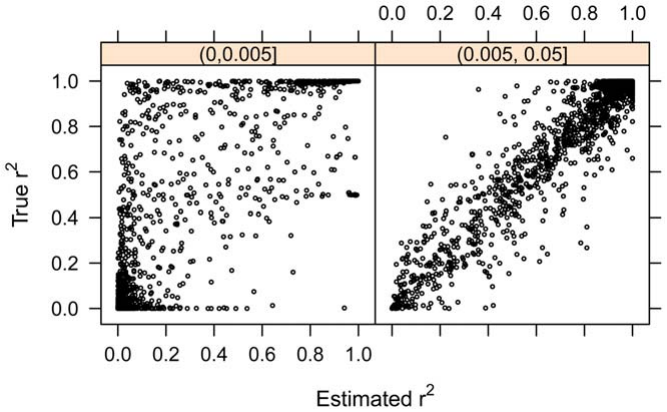
True r^2^ versus the estimated r^2^. True r^2^, squared correlation of the true allele dosage (based on genotypes derived from DeepSeq Variant Set), is plotted versus the estimated r^2^, the ratio of the variance of the imputed allelic dosage and the variance of the true allelic dosage assuming Hardy- Weinberg equilibrium for each imputed variant, for the reference set consisting of 3713 samples with Affymetrix 500K GWAS data. Very rare variants, MAF≤0.005, (1331 variants) and more common variants, MAF>0.005, (1776 variants) are shown separately. These results illustrate that while the estimated r^2^ calculated for imputed markers reflects the true r^2^ obtained when genotype data is available for common and rare variants, it is likely to under estimate the true r^2^ for very rare variants.

Figure 1 plots the estimated r^2^ versus true r^2^ for the Affymetrix 500K dataset with 3713 reference samples for variants with MAF no greater or greater than 0.005 which removes almost all variants with poorly calibrated estimated r^2^ values. This plot is shown for Affymetrix 500K data set with 3713 reference samples, but the results for all other datasets are similar. The Pearson correlation coefficients relating true and estimated r^2^ for variants with MAF≤0.005 were 0.787, 0.825, 0.783 for Affymetrix 500K data set with n = 3713, Affymetrix 6.0 data set with n = 562, and Illumina 550K data set with n = 3713 reference samples, respectively. The Pearson correlation coefficients relating true and estimated r^2^ for these same reference sets for variants with 0.005<MAF≤0.5 were 0.951, 0.971, and 0.983. These results illustrate that the estimated r^2^ calculated for imputed markers reflects the true r^2^ obtained when genotype data is available making the reported estimated r^2^ a valuable metric of imputation quality for markers with MAF greater than 0.005. Estimated r^2^ is likely to under estimate true r^2^ for extremely rare variants (MAF≤0.005). We also note that this correlation was stronger in our smaller, more ethnically homogeneous reference sets (n≤1200) as opposed to our largest reference sets (n = 3713).

**Figure 2:**
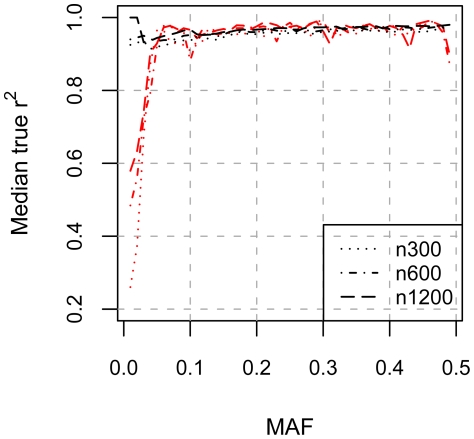
Median true r^2^ for array and exon-derived variants in MAF bins. The median true r^2^ for 0.01≤MAF≤0.5 for 300, 600, and 1200 reference samples, derived from imputing Illumina 550K SNPs not available on the Affymetrix 500K platform (shown in black), and median true r^2^ for 300, 600, and 1200 reference samples for Affymetrix 500K platform, calculated using DeepSeq Variant Set (shown in red), are plotted versus minor allele frequency bins. Bins were defined to be centered on MAFs ranging from [0.01, 0.49] with a total width of 0.02. The median true r^2^ values for these datasets are very similar except for variants with MAF≤0.05 illustrating that imputation using reference samples with variants from the exons and flanking regions of genes is similar to more uniformly spaced markers.

For common SNPs, the impact of a reference sample with variants from the exons and flanking regions of genes rather than more uniformly spaced as would be available in HapMap 3 [2], can be illustrated by comparing our median true r^2^ to those obtained from imputing SNPs for each Illumina 550k SNP present in the HapMap but not available on the Affymetrix 500K panel. Figure 2 plots median true r^2^ for variants with 0.01≤MAF≤0.5 for 300, 600, and 1200 reference samples in Europeans [[ along with median true r^2^ for 300, 600, and 1200 reference samples for the Affymetrix 500K platform calculated using variants from the DeepSeq Variant Set that were matched based on MAF to their variants. The median true r^2^ values for these datasets are very similar except for variants with MAF≤0.05 illustrating that imputation using reference samples with variants from the exons and flanking regions of genes is similar to more uniformly spaced markers. For variants with MAF≤0.05, the median true r^2^ for the variants identified by sequencing is much lower than that for variants that are on the Illumina 550K panel which were chosen because they are good proxies for other SNPs [2].

**Figure 3:**
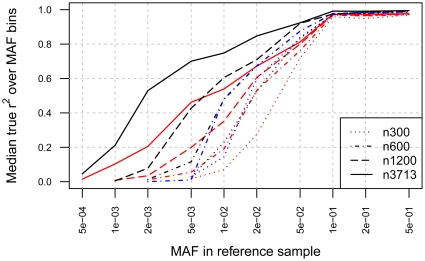
Median true r^2^ versus MAF bins. The median true r^2^ for the MAF bins of (0,0.0005], (0.0005, 0.001], (0.001, 0.002], (0.002, 0.005], (0.005, 0.01], (0.01, 0.02], (0.02, 0.05], (0.05, 0.1], (0.1, 0.2], and (0.2, 0.5] are plotted versus MAF in the reference sample for the DeepSeq Variant Set. Reference set samples sizes of 300, 600, 1200, and 3713 were available for both Affymetrix 500K (shown in red) and Illumina 550K (shown in black) platforms. Reference set sizes of 300 and 562 were available for the Affymetrix 6.0 platform (shown in blue). Median true r^2^ values are near to 1.0 for variants with MAF≥0.1. For variants with MAF<0.1, the number of reference samples and the GWAS platform both influence the median true r^2^.

Figure 3 plots the median true r^2^ calculated for MAF bins versus MAF in the reference sample for all variants present in the DeepSeq Variant Set. For variants with 0.05<MAF≤0.5, the number of reference samples and the GWAS platform have a minimal effect on the median true r^2^, even when the reference panel size is 100 (data not shown). For variants with 0.01<MAF≤0.05, the number of reference samples begins to have more of an effect but in this frequency range the GWAS platform is more influential. In this frequency bin, the median true r^2^ values are 0.80, 0.80 and 0.62 for reference sample sets of 600 (562 for Affymetrix 6.0) versus 0.73, 0.72 and 0.48 for reference sample sets of 300 for the Illumina 550K, Affymetrix 6.0 and Affymetrix 500K panels, respectively. For variants with MAF≤0.01, both number of reference samples and the GWAS platform influence the median true r.^2^ The median true r^2^ for variants with 0.005<MAF≤0.01 for 300 reference samples ranges from 0.07 to 0.23 depending on the GWAS platform but for 600 reference samples, it ranges from 0.19 to 0.48. This illustrates the importance of larger reference samples for imputing variants with MAF≤0.01. For variants with 0.005<MAF≤0.01, the median true r^2^ values for a reference sample size of 600 are 0.48 for the Illumina 550K and Affymetrix 6.0 panels versus 0.19 for the Affymetrix 500K panel. For reference panels with greater than approximately 600 samples when imputing variants with 0.005<MAF≤0.01, utilizing the Affymetrix 500K panel results in median true r^2^ values about 0.2 lower than those for the Illumina 550K panel.

**Figure 4:**
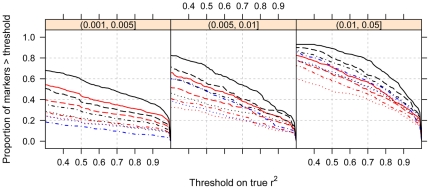
Cumulative distribution function of true r^2^. The cumulative distribution function, the proportion of markers with a true r^2^ greater than the threshold, is shown for all reference sample sizes and GWAS platforms described in Figure 3. The proportion of well imputed variants decreases as the variant MAF decreases.

Figure 4 shows the cumulative distribution function of the true r^2^ for different reference sample sizes for variants with 0.001<MAF≤0.005, 0.005<MAF≤0.01, and 0.01<MAF≤0.05. For variants with 0.001<MAF≤0.005, the proportion of well imputed SNPs (true r^2^>0.7) was approximately 48% for the Illumina 550K data set with n = 3713 reference samples and less than 35% for the remaining reference sets. Comparing the three panels in Figure 4 illustrates that reference sample size has more of an influence than GWAS panel as the MAF of the variant decreases. Furthermore, the proportion of well imputed variants remains approximately 54% for the Illumina 550K data set with n = 3713 reference samples even for variants with 0.005<MAF≤0.01. For common SNPs with MAF>0.05, greater than 86% of these SNPs can be well imputed (true r^2^>0.7) when using GWAS platforms that rely on tagging SNPs or the largest reference panel for the Affymetrix 500K data set with n = 3713 reference samples, data not shown.

## Discussion

We characterized the performance of genotype imputation with reference panels of 100 to 3713 subjects for variants with spacing that would be representative of extending our evaluation of variants identified via exon-targeted sequencing into additional samples for three GWAS panels. Even with reference sample sets of 100 subjects, greater than 80% of variants with MAF>0.05 can be well imputed (estimated r^2^>0.7). For variants with 0.01<MAF<0.05, the proportion of well imputed variants is dependent on the number of reference samples and the GWAS panel but it can be as high as 50–70% for reference panels larger than 1200. An appreciable number of variants with MAF≤0.005, can be well imputed with reference panels including 3713 subjects and more comprehensive GWAS panels (i.e. 48% of variants with 0.001≤MAF≤0.005 for the Illumina 550K platform will be well imputed with a reference panel of 3713 individuals). We investigated how missing data in the reference panel (from the DeepSeq Variant Set) affected the estimated r^2^ which revealed that missing rates less than 30% have little impact on the distribution of estimated r^2^ values (data not shown).

Our comparisons of imputing markers with uniform spacing versus imputing markers derived from the DeepSeq Variant Set showed no discernable differences for markers with MAF>0.05. These results are informative with respect to what can be expected for imputation with HapMap3 data where the SNP density is higher and the number of samples of European ethnicity is now 205 [2]. For moderately rare SNPs (0.01<MAF≤0.05), the median true r^2^ values are lower, similar to results found in analysis of HapMap 3 and ENCODE data that showed that array SNPs, especially those selected on the basis of LD, are much more likely to be good proxies for each other in comparison with newly discovered variants. We did not compare these results to genotyping imputation using the 1000 Genomes dataset as a reference panel since the current 1000 Genomes dataset is derived from low depth sequencing data.

This analysis provides one of the first evaluations of imputation for variants identified in the exons and flanking regions of genes, especially for variants with MAF less than or equal to 0.05. Deriving variants from high depth sequence data enabled us to evaluate imputation performance by comparing imputed SNPs to variants at very low frequencies which are generally unreliably genotyped using other assay methods. For moderately rare SNPs, 0.01<MAF≤0.05, the GWAS platform was an important factor for high quality imputation. The Illumina 550K GWAS platform includes tag SNPs derived from greater than 2 million common SNPs genotyped in HapMap Phase 2 data [9] which were selected after sequencing a few individuals so that rare haplotypes are not well-represented. The Affymetrix 500K platform includes SNPs selected on the basis of sequence constraints when choosing the probes and additional tag SNPs were added to form the Affymetrix 6.0 array. Therefore, it is not surprising that imputation performance is better for GWAS platforms that focused on SNPs that are good proxies for nearby SNPs. Analysis of the relationship between maximum pairwise R^2^_LD_ for any SNP within 1 Mb and true r^2^, observed for that SNP showed that for common markers, SNPs that are well tagged by GWAS panels will almost always be well imputed but being well tagged by a single marker from the GWAS panel is not a requirement for high imputation quality. This suggests that higher order linkage disequilibrium provides information on untyped variants and contributes to the imputation quality. For rare and very rare SNPs, 0<MAF≤0.01, the number of reference samples becomes more of a factor since observing multiple copies of a SNP is important for establishing the haplotype background for observed SNPs. Figure 3 provides the median true r^2^ value which summarizes imputation quality but fails to convey the distribution of true r^2^ values for each MAF bin. For almost all reference set sizes and GWAS platforms, the distribution of true r^2^ values is negatively skewed for MAFs greater than 0.01 and positively skewed for MAFs less than 0.01.

Our study provides a description of imputation performance for multiple GWAS panels using reference panels up to 3713 subjects with variants derived from high depth sequence data from the exons and flanking regions of genes. Very rare variants (MAF<0.005) are unlikely to be imputable without reference panels with greater than 1200 subjects, while almost all common SNPs (MAF≥0.05) and approximately 40% of rare variants (0.005<MAF<0.05) for reference panel sizes of 1200 individuals or more will be imputable. Therefore, genotype imputation into additional samples with GWAS data will increase the sample size available for genotype-phenotype analysis for common and moderately rare variants with performance depending on the reference panel size for very rare variants (MAF<0.005).

## Materials and Methods

### Ethics Statement

GSK collected human blood in collaborative research trials with investigators during 2002–2010 for other studies. Written informed consent was obtained and recorded via electronic case report form. The consents allowed for continued or future evaluation of variants associated with diseases. The work described in this manuscript represents a re-use of these samples and data and no new human interventions were conducted. Therefore this research involves the study of existing samples and data. No additional IRB approvals were sought for this specific portion of the work. The names of all ethics committee/institutional review boards that approved the original protocols for sample collection include: Committee on Human Research, University of California, San Francisco, Ethics Committee of Basel City and Canton, Basel University Hospital, Medical Ethics Committee University Medical Centre, Amsterdam for the Multiple Sclerosis gene MSA collection; Committee on Ethics in Clinical Research, CHUV, Lausanne University, Lausanne, Switzerland for the CoLaus collection; Regional Committee for Medical Research Ethics (REKIII), Faculty of Medicine, University of Bergen, Norway for the GenKOLs collection, Ethics Committee Multicentre Trials, Bulgaria, McGill University Health Center Research Ethics Board, Montreal, Canada, Office of Research Services Clinical Research Ethics Board, University of British Columbia, Canada, Capital Health Research Ethics Board, Halifax, Canada, Hamilton Health Sciences/Faculty of Health Sciences Research Ethics Board, Hamilton, Ontario, Canada, Providence Health Care Research Institute, Vancouver, British Columbia, Canada, Comité d'éthique de la recherche, Québec, Canada, Queens University Office of Research Services, Kingston, Ontario, Canada, Multicentric Ethics Committee Fakultni nemocnice v Motole, Prague, Czech Republic, Den videnskabsetiske komité for region hovedstaden, METC Zuidwest-Holland, MHHA Kirkels-Breukers, Delft, The Netherlands, Regional Ethic Committee West, Haukeland University Hospital, Bergen, Norway, The National Medical Ethics Committee of the Republic of Slovenia, Ljubljana, Slovenia, Comité ètic d'investigació clínica Illes Balears, Palma de Mallorca, Spain, Oxfordshire REC C, Bicester, United Kingdom, MD Human Subjects Committee, Torrance, California, USA, Research/Human Subjects Committee, St Elizabeth's Medical Center, Boston, Massachusetts, US, Goodwyn Institution Review Board, Cincinnati, Ohio, US, Western International Review Board, Olympia, Washington, US, Baylor College of Medicine IRB, Houston, Texas, US, Committee for the Protection of Human Subjects, Dartmouth-Hitchcock Medical Center, Hanover, New Hampshire, US, National Jewish Medical & Research Center IRB, Denver, Colorado, US, University of Nebraska Medical Center IRB, Omaha, Nebraska, US, Yale University School of Medicine Human Investigation Committee, New Haven, Connecticut, US, Mayo Foundation IRB, Rochester, Minnesota, US, Creighton University Medical Center IRB, Omaha, Nebraska, University of Pittsburgh IRB, Pittsburgh, PA, US, Brigham & Women's Hospital IRB, Boston, Massachusetts, US, John Hopkins School of Medicine, Baltimore, MD, St Francis Hospital & Medical Center IRB, Hartford, Connecticut, US for the ECLIPSE Study; MedStar Research Institute Institutional Review Board, Washington Health Center Research Committee for the Coronary Artery Disease Medstar study; Bayerische Landesärztekammer (Bavarian Ethics Committee) for the Unipolar Depression Study; University Research Ethics Committee of the University of Dundee, The Joint South London and Maudsley and The Institute of Psychiatry NHS Research Ethics Committee London, UK, Center for Addiction and Mental Health Research Ethics Board, Toronto, Canada for the Bipolar Disorder Study; MREC for Scotland, Edinburgh, Scotland, Health Sciences Research Ethics Committee, Laval University, Canada, Ethics Committee of the Medical Faculty of Ludwig Maximilian University, Munich, Germany for the Schizophrenia study, University of Western Ontario Research Ethics Board for Health Sciences Research Involving Human Subjects, Ontario, Canada, Centre Hospitalier regional de Trois-Rivieres comite d'ethique de la recherché, Trois-Rivieres, Quebec, Canada, Centre for Addiction and Mental Health Research Ethics Board, University of Toronto, Toronto, Canada, University of Western Ontario Research Ethics Board for Health Sciences Research Involving Human Subjects, Ontario, Canada, SCO Health Service Research Ethics Board, Ottawa, Ontario, Canada, University Health Network Research Ethics Board, Toronto, Canada, University of British Columbia Clinical Research Ethics Board, Vancouver, British Columbia, Canada, Douglas Hospital Research Ethics Board, Montreal, Quebec, Canada for the Alzheimer's Disease GenADA study; Committee on Human Research, University of California-San Francisco, California, US, Royal Adelaide Hospital Research Ethics Committee, University of Texas Southwestern Medical Center at Dallas Institutional Review Board, Dallas, Texas, US, Human Research Ethics Board of the University of Ottawa Heart Institute, Ottawa, Ontario, Canada, and University of Lausanne, Ethics Committee, Lausanne, Switzerland for the Metabolic Syndrome GEMS study, Regional Committee for Medical Research Ethics, Sør Health Region, Oslo Norway for the Epilepsy HiTDIP study; and Cantonal Ethics committee of the Canton of Zurich, Specialized Sub-Committee for Psychiatry, Neurology, Neurosurgery, Zurich, Switzerland for the Epilepsy GenEpa study.

Analyses were carried out using 8865 subjects for whom we had genotype data derived from high-depth sequencing data and commonly used GWAS panels to characterize genotype imputation for variants found in the exons and flanking regions [10]–[20].

The high-depth of sequence, including 850 kb sequence data used in this experiment, were generated by BGI (Shenzhen, China) by sequencing the exons plus 50 bp of flanking sequence of 202 genes resulting in approximately 8351 kb of coding and 323 kb of noncoding (untranslated) exons. Candidate variants were identified for each sample where a genotype was called with a minimum sequencing depth of four, a minimum consensus quality of 20 with no other variants within four base pairs. Genotypes were called in all samples for all variant positions identified by aggregating all sequenced samples. Consensus genotypes were called at these position bases for each sample with a minimum depth of seven and minimum consensus quality of 20. The median sequence depth per sample was 27×. Samples were excluded from the data if 1) their average sequencing depth was less than 10, 2) sequence-based genotypes were more than 15% discordant with genome-wide panel genotypes or 3) the sample was sequenced multiple times and had lower average sequencing depth. Analysis of 133 sample duplicates resulted in a discordance rate among heterozygous genotype calls of 0.90% with lower rates in more common variants. An overall heterozygote genotype error rate was estimated to be 0.50%. These quality settings resulted in median genotype missingness of 0.0069, 0.011, 0.014, and 0.021 for variants with MAF≤0.001, 0.001<MAF≤0.005, 0.005<MAF≤0.05, MAF>0.05, respectively. In contrast to other genotyping platforms, subject-level genotype missing rates were not correlated with genotype accuracy. We removed variants with a missing rate greater than 30% and singleton variants since they cannot be phased for imputation. This “DeepSeq Variant Set” included 9077 variants.

Our sample consisted of 8865 samples with sequence data as described above and GWAS data for one of the following platforms: Affymetrix 500K, (n = 3983), Illumina 550K (n = 4309) and Affymetrix 6.0 (n = 573). Due to the large number of sequenced samples in our experiment, we partitioned these sequenced samples into reference sets and “to-be-imputed” study sets. We used the densely genotyped reference panel to predict unobserved genotypes in the study sample using genotype imputation. Then we compared the imputed genotypes to genotypes in the DeepSeq Variant Set. Table 1 lists the reference set and study set sizes evaluated. We selected reference set sizes of 100, 300, 600, 1200 and 3713 to enable comparisons with [8]; [21] for uniformly spaced variants and between the Affymetrix 500K and Illumina 550K platforms. The reference set size of 562 (see Table 1) for the Affymetrix 6.0 platform was the result of partitioning the sequenced samples into reference (98% of the overall sample) and study samples (2% of the overall sample) multiple times holding out study samples, sequentially, so that we developed a set of imputed genotypes for each sample. For each variant in the DeepSeq Variant Set, we also calculated pairwise R^2^ (due to linkage disequilbrium – R^2^_LD_) with all SNPs within 1 Mbp on each GWAS panel.

Genotype imputation analysis was carried out using BEAGLE with the default settings. All GWAS SNPs within 1 Mbp of the sequenced regions were included in the analysis. When genotypes were missing in the reference panel, they were imputed based on the available data similarly to missing data in the study sample. We calculated the true r^2^ or the squared correlation of the true allele dosage (based on genotypes derived from DeepSeq Variant Set) and the imputed allele dosage for each dataset [8]. This measure quantifies the similarity of the imputed allele dosage with the dosage based on genotypes. We also calculated the estimated r^2^ (MACH's ratio of variances metric), the ratio of the variance of the imputed allelic dosage and the variance of the true allelic dosage assuming Hardy- Weinberg equilibrium for each imputed variant [7]. This is a metric of expected imputation quality.

To compare the imputation quality of BEAGLE and minimac, we imputed chromosome 1 data using the Affymetrix 500K reference set of 3713 subjects with 270 study subjects using both programs. Minimac imputation was run using 10 rounds and 300 states as parameters. Running times were 310 minutes for BEAGLE and 160 minutes for minimac, respectively. We compared true r^2^, estimated r^2^, mean genotypic error rate (among the variants), and mean allelic error rate (among variants). The allelic or genotypic error rate is the proportion of allelic or genotypic mismatches among all allelic or genotypic comparisons.

## Supporting Information

Figure S1 **Cumulative distribution functions of the true r^2^ for minimac and BEAGLE.** The cumulative distribution function, the proportion of markers with an estimated r^2^ greater than the threshold, is shown for reference sample size of 3713 for chromosome 1 markers for the Affymetrix 500K platform. Imputation performance is similar for the two analysis methods.
(TIF)

Information S1 (DOC)
