# Supplementary material for: Performance of Genotype Imputation for Rare Variants Identified in Exons and Flanking Regions of Genes
Source: PLoS One. 2011 Sep 19;6(9):e24945. doi: 10.1371/journal.pone.0024945 (PMC3176314; doi:10.1371/journal.pone.0024945)
Supplement: Information S1 — (DOC) [file pone.0024945.s002.doc]

**Supplementary Information for**

**Performance of Genotype Imputation for Rare Variants Identified in Exons and Flanking Regions of Genes**

Li Li, Yun Li, Sharon R. Browning, Brian L. Browning, Andrew J. Slater, Xiangyang Kong, Jennifer L. Aponte, Vincent E. Mooser, Stephanie L. Chissoe, John C. Whittaker, Matthew R. Nelson, Margaret Gelder Ehm

# Study sample

A description of the subjects included in the QPOC DeepSeq Variant set analyzed for this paper are described in further detail below. The primary selection criteria applied to each study was the availability of at least 10ug of DNA with a concentration at least 195 ng/ul from a primary blood sample.

## Study descriptions

**CoLaus Study**: A population-based study of 6,188 European white subjects age 35-75 years drawn from Lausanne Switzerland, through the CHUV University Hospital [1].  Subjects included in the current study include 1,774 participants in the follow-on study of psychiatric traits (PsyCoLaus) [2] and 772 extremes of several selected cardiovascular disease-associated traits. There was an overlap of 460 subjects between these two selections.

**Metabolic Syndrome GEMS Study**: The GEMS Study of Metabolic Syndrome and related traits included two types of samples; families and a set of unrelated cases and controls. Families (3384 individuals from 535 families) were recruited from six study sites located in Australia, Canada, Finland, Switzerland, Turkey and the United States. Eligible families consisted of a minimum of two siblings (an affected sib-pair) with atherogenic dyslipidemia (ADL). In the case-control arm, a set of approximately 1,000 cases with ADL and 1,000 normolipidemic controls were recruited from the same GEMS sites. Details of the recruitment procedures, subject characteristics, and inclusion/exclusion criteria for both the family and case control studies have been previously described [3,4]. The current study includes 1,570 unrelated cases and controls and 30 parent-offspring trios for assessing sequence data quality.

**Coronary Artery Disease (CAD) MedStar Study**: A premature CAD collection designed to investigate the genetics of plaque stability in acute coronary syndrome (ACS). The full study is comprised of 452 ACS CAD cases, 491 non-ACS CAD cases, and 483 non-CAD controls [5]. Subjects were identified prospectively from the patient population of Cardiovascular Research Institute (MedStar/Washington Hospital Center). Standard criteria were used to identify cases with myocardial infarction and cases diagnosed with clinically significant coronary atherosclerosis without myocardial infarction. Subjects included in the current study include a selection of 609 ACS and non-ACS CAD cases.

**Multiple Sclerosis geneMSA Study**: A study of 1005 thoroughly phenotyped multiple sclerosis (MS) cases and 1,012 matched controls primarily of European ancestry from three sites in the United States, the Netherlands and Switzerland [6]. The current study includes 673 cases.

**Alzheimer’s Disease genADA Study**: Study includes individuals with Alzheimer’s disease (AD) diagnosed by the National Institute of Neurological and Communicative Diseases and Stroke/ Alzheimer’s Disease and Related Disorders Association criteria. Subjects were recruited from nine memory referral clinics in Canada [7]. The current study includes 705 cases.

**Unipolar Depression Study**: A study of 1,000 cases recruited from three ascertainment sites in Southern Germany (Munich, in Augsburg and in Ingolstadt) and 1,029 controls ascertained by the Max Plank Institute of Psychiatry in Munich. Cases diagnosed with recurrent major depressive disorder and controls were age and gender-match non-affected controls [8]. The current study includes 775 cases.

**Schizophrenia Study**: A study of approximately 1,600 cases and 850 controls collected from four sites in Aberdeen, UK, Greenock, UK, Munich, Germany and Quebec City, Canada. Cases were diagnosed with schizophrenia according to DSM-IV or ICD-10 criteria and healthy volunteers were randomly selected from the general population [9]. The current study includes 1,109 cases.

**Bipolar Disorder Study**: A study of 965 bipolar cases and 933 controls from a multicenter study subjects of European ancestry from three different sites the Centre for Addiction and Mental Health in Toronto, Canada, the Institute of Psychiatry in London, UK and the University of Dundee, UK. Each case was assessed when euthymic and had to have been diagnosed (lifetime) with the DSM-IV/ICD-10 bipolar I or bipolar II disorder [9]. The current study includes 786 cases.

**Epilepsy HitDIP Study**: A study of 719 cases and 687 controls recruited from Norway and Finland. All patients had a definite diagnosis of epilepsy according to International League Against Epilepsy (ILAE) definitions. Controls had no neuropsychiatric condition[10]. The current study includes 185 Finnish cases.

**Epilepsy GenEpa Study**: A study of 318 cases and 348 controls from Swiss Epilepsy Centre, Zurich [10,11]. All patients had a definite diagnosis of epilepsy according to ILAE definitions. Controls had no neuropsychiatric condition. The current study includes 125 cases.

**Chronic Obstructive Pulmonary Disease ECLIPSE Study**: ECLIPSE (Evaluation of COPD Longitudinally to Identify Predictive Surrogate End-points) is a three-year non-interventional longitudinal prospective study being conducted at 46 centers in 12 countries and is comprised of clinically relevant COPD individuals with Global Initiative for Chronic Obstructive Lung Disease (GOLD) stage 11_IV COPD with a number of smoking and non-smoking and non-disease controls [12]. The current study includes 1,002 cases from ten countries.

**COPD genKOLs Study**: A study of approximately 1,000 cases and 1,000 controls from Bergen, Norway. Cases consist of α1-antitrypsin deficiency-negative individuals with moderate to severe COPD according to GOLD criteria. [13]. The current study includes 782 cases.

# References

Reference List

1. Firmann M, Mayor V, Vidal PM, Bochud M, Pecoud A et al (2008) The CoLaus study: a population-based study to investigate the epidemiology and genetic determinants of cardiovascular risk factors and metabolic syndrome. BMC Cardiovasc Disord 8: 6-

2. Preisig M, Waeber G, Vollenweider P, Bovet P, Rothen S et al (2009) The PsyCoLaus study: methodology and characteristics of the sample of a population-based survey on psychiatric disorders and their association with genetic and cardiovascular risk factors. BMC Psychiatry 9: 9-

3. Ling H, Waterworth DM, Stirnadel HA, Pollin TI, Barter PJ et al (2009) Genome-wide linkage and association analyses to identify genes influencing adiponectin levels: the GEMS Study. Obesity (Silver Spring) 17: 737-744.

4. Wyszynski DF, Waterworth DM, Barter PJ, Cohen J, Kesaniemi YA et al (2005) Relation between atherogenic dyslipidemia and the Adult Treatment Program-III definition of metabolic syndrome (Genetic Epidemiology of Metabolic Syndrome Project). Am J Cardiol 95: 194-198.

5. Assimes TL, Holm H, Kathiresan S, Reilly MP, Thorleifsson G et al (2010) Lack of association between the Trp719Arg polymorphism in kinesin-like protein-6 and coronary artery disease in 19 case-control studies. J Am Coll Cardiol 56: 1552-1563.

6. Baranzini SE, Wang J, Gibson RA, Galwey N, Naegelin Y et al (2009) Genome-wide association analysis of susceptibility and clinical phenotype in multiple sclerosis. Hum Mol Genet 18: 767-778.

7. Li H, Wetten S, Li L, St Jean PL, Upmanyu R et al (2008) Candidate single-nucleotide polymorphisms from a genomewide association study of Alzheimer disease. Arch Neurol 65: 45-53.

8. Muglia P, Tozzi F, Galwey NW, Francks C, Upmanyu R et al (2010) Genome-wide association study of recurrent major depressive disorder in two European case-control cohorts. Mol Psychiatry 15: 589-601.

9. Francks C, Tozzi F, Farmer A, Vincent JB, Rujescu D et al (2010) Population-based linkage analysis of schizophrenia and bipolar case-control cohorts identifies a potential susceptibility locus on 19q13. Mol Psychiatry 15: 319-325.

10. Heinzen EL, Radtke RA, Urban TJ, Cavalleri GL, Depondt C et al (2010) Rare deletions at 16p13.11 predispose to a diverse spectrum of sporadic epilepsy syndromes. Am J Hum Genet 86: 707-718.

11. Kasperaviciute D, Catarino CB, Heinzen EL, Depondt C, Cavalleri GL et al (2010) Common genetic variation and susceptibility to partial epilepsies: a genome-wide association study. Brain 133: 2136-2147.

12. Vestbo J, Anderson WH, Coxson HO, Crim C, Dawber F et al (2008) Evaluation of COPD Longitudinally to Identify Predictive Surrogate End-points (ECLIPSE). Eur Respir J 31: 869-873.

13. Pillai SG, Ge D, Zhu G, Kong X, Shianna KV et al (2009) A genome-wide association study in chronic obstructive pulmonary disease (COPD): identification of two major susceptibility loci. PLoS Genet 5: e1000421-
